# Attaching metabolic expenditures to standard occupational classification systems: perspectives from time-use research

**DOI:** 10.1186/s12889-017-4546-7

**Published:** 2017-07-03

**Authors:** J. Deyaert, T. Harms, D. Weenas, J. Gershuny, I. Glorieux

**Affiliations:** 1 Research group TOR, Department of Sociology, Vrije Universiteit Brussel, Pleinlaan 2, 1050 Brussels, Belgium; 2 Centre for Time Use Research (CTUR), Department of Sociology, University of Oxford, 74 Woodstock Road, Oxford, OX2 6HP UK

**Keywords:** Time-use data, Physical activity (PA), Occupational activity (OA), Physical activity energy expenditure (PAEE), Standard occupational classification systems, Isco 2008, Methodology

## Abstract

**Background:**

Traditionally, time-use data have been used to inform a broad range of economic and sociological research topics. One of the new areas in time-use research is the study of physical activity (PA) and physical activity energy expenditure (PAEE). Time-use data can be used to study PAEE by assigning MET values to daily activities using the Ainsworth Compendium of Physical Activities. Although most diarists record their daily activities accurately and in detail, they are only required to record their paid working hours, not the job-specific tasks they undertake. This makes it difficult to assign MET values to paid work episodes.

**Methods:**

In this methodological paper, we explain how we addressed this problem by using the detailed information about respondents’ occupational status included in time-use survey household and individual questionnaires. We used the 2008 ISCO manual, a lexicon of the International Labour Organization of occupational titles and their related job-specific tasks. We first assigned a MET value to job-specific tasks using the Ainsworth compendium (2011) then calculated MET values for each of the 436 occupations in the ISCO-08 manual by averaging all job-specific MET values for each occupation.

**Results:**

The ISCO-08 Major Groups of ‘elementary occupations’ and ‘craft and related trades workers’ are associated with high PAEE variation in terms of their job-specific MET values and together represented 21.6% of the Belgian working population in 2013. We recommend that these occupational categories should be prioritised for further in-depth research into occupational activity (OA).

**Conclusions:**

We developed a clear and replicable procedure to calculate occupational activity for all ISCO-08 occupations. All of our calculations are attached to this manuscript which other researchers may use, replicate and refine.

**Electronic supplementary material:**

The online version of this article (doi:10.1186/s12889-017-4546-7) contains supplementary material, which is available to authorized users.

## Background

Regular physical activity (PA) has a preventative effect on a number of non-communicable diseases and health conditions [1–3]. Although many studies report that leisure time physical activity (LTPA) has beneficial health outcomes for all workers irrespective of their occupational workload [4, 5], there is more controversy about the health benefits associated with occupational activity (OA) [6–8].

Health researchers mostly assess physical activity energy expenditure (PAEE) using reported or objective ‘device-based’ methods [9]. The self-reported measures used in epidemiological research (e.g. the International Physical Activity Questionnaire (IPAQ) or the Global Physical Activity Questionnaire (GPAQ)) are used in a standardised and validated format worldwide with large population representative samples. However, they carry limitations including problems of recall and social desirability bias and further, often focus on single life domains [10]. Recent studies have used objective data collection methods and devices (e.g. wearable cameras, accelerometers, smart watches, global positioning systems (GPS)) to capture PAEE. These instruments provide precise and accurate measurements of specific types of PA, but include little or no information about the context in which the activities took place. Furthermore, due to time and cost constraints compared with self-report methods, studies using device-based methods usually have small samples [9, 10].

### Time-use and physical activity research

It is only recently that health researchers have begun to use data from time-use surveys to study PAEE [11]. Time-use data are collected worldwide by statistical institutes and universities, and used for a wide range of research applications, mainly in the fields of economics and sociology. Given the richness and availability of international time-use data (e.g. the Multinational Time-Use Study (MTUS) [12]), scholars from other disciplines have become interested in using them to inform their research. One of the advantages of time-use data is that they provide a continuous record across each 24-h period so they can inform all of the life domains in which health researchers are interested. Therefore, these data can annotate and contextualise device-based data and enable analysts to calculate the time respondents spend in activities of different intensities across all of the life domains (i.e. OA, LTPA, travel, housework, childcare, home maintenance and sleep). This contributes to a deeper understanding of PAEE within and between domains such as OA, LTPA and sedentary behaviour (SB).

#### Time-use studies

**Fig. 1:**
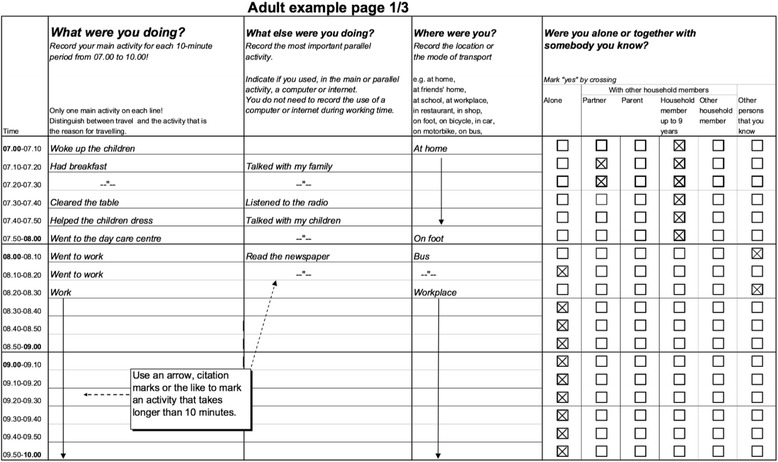
Extract of a written diary following the HETUS guidelines [13]

In time-use studies, respondents keep track of their daily activities using a self-report diary. Diarists record in their own words the main (primary) activity in which they were engaging; any activities they were doing at the same time (secondary/simultaneous); who was present during this activity (with whom/co-presence) and; where the activity took place (location) or if travelling, the mode of transport (walking, cycling, driving, public transport, etc.). The Harmonized European Time-Use Survey (HETUS) Guidelines, used by statistical agencies and research institutes across more than 20 European countries, recommends a ‘tomorrow’ diary where respondents keep a continuous record of their activities in a paper-and-pencil or electronic (computer or app) form [13]. Respondents record their activities for two 24-h periods (04:00 to 04:00) – one randomly selected week and weekend day. Starting from the respondents’ own words, trained coders assign activity codes to the primary and secondary activities using the HETUS Activity Coding Lexicon [13]. The continuous and sequential recording used in time-use studies covers all the activities in which respondents engage throughout the day – in contrast with the un- contextualised behaviour-specific approach of recall questionnaires such as the IPAQ or GPAQ. Therefore, time-use surveys generate more valid and reliable data than questionnaires that only focus on specific daily activities [14]. The sequential recording required for diaries makes it difficult for respondents to manipulate subsequent activities (e.g. substituting watching TV for physical exercise), which lowers social desirability bias and measurement error. Figure 1 presents an example of a coded diary following the HETUS Guidelines.

In the majority of time-use studies, respondents complete individual and household questionnaires, which provide detailed occupational and socio-demographic information; recent time-use surveys ask respondents to rate their subjective health and well-being, provide self-estimated height and weight and record levels of enjoyment or stress associated with daily activities.

#### Time-use and physical activity energy expenditure (PAEE)

Tudor-Locke and colleagues [11] pioneered a study linking the Activity Coding Lexicon from the 2003 American Time-Use Survey (ATUS) with the Ainsworth Compendium of Physical Activities (hereafter ‘Compendium’) [15, 16] to study PA levels in US adults. In a small-scale validation study of Australian blue-collar workers, van der Ploeg and colleagues found relatively high correlations between PA inferred from time-use diaries and objectively-measured accelerometer data [17]. They concluded that time-use survey data appeared to be more valid for non-occupational PA population surveillance than more traditional self-reported surveillance systems. Since then, several studies using time-use data to calculate PAEE have been carried out [18–22]. All these studies used the Compendium to assign MET values to time-use diary data.

Ainsworth and colleagues developed the Compendium to create a comprehensive and standardised list of different types of daily activities and their corresponding measure of PAEE, expressed in Metabolic Equivalent of Task (hereafter ‘MET values’ or ‘METs’). First published in 1993 and updated in 2000 and 2011, it consists of 821 different daily activities grouped into 21 major headings [15, 16, 23]. Where it was not possible to assign MET values to specific activities based on laboratory or field studies, experts in the assessment of PA calculated estimates for similar types of activities. The MET values in the Compendium apply to able-bodied adults aged 18–65 years, but cannot estimate the precise energy cost of PA for individuals which vary according to body mass, adiposity, age, sex, efficiency of movement, and the geographic and environmental conditions in which the activities took place [15]. The MET values in the Compendium represent the ratio of the work metabolic rate to the standard resting metabolic rate and so indicate how physically demanding an activity is compared to a situation at rest. One MET is defined as 1 kcal/kg/h and is roughly equivalent to the energy cost of sitting quietly. A MET is also defined as oxygen uptake in ml/kg/min, with one MET equal to the oxygen cost of sitting quietly, equivalent to 3.5 ml/kg/min [23]. Intensity categories are broadly defined as light (<3 METs), moderate (3–6 METs) and vigorous (>6 METs); light-intensity categories can be interpreted as sleeping activities (<1 MET) or sedentary/lying/sitting activities (≥1 and <3 METs) [11].

Although diarists record their daily non-work activities in detail, they are only required to register the hours they spent in paid work rather than the job-specific tasks they undertook during the working day, which makes it difficult to calculate robust estimates of OA using time-use data alone. Below, we explain how we overcame this problem by assigning MET values to work episodes based on the respondent’s occupation included in the household and individual questionnaires associated with time-use studies. Because there is a lack of replicable objectively-measured data on OA that can be linked to occupational classification systems, we developed a procedure and set of calculations that can be used by health researchers to link occupational PAEE measures to existing surveys (i.e. to provide details about work episodes in time-use data), and to inform future research into the objective measurement of OA.

## Methods

The degree of detail in time-use diary Activity Coding Lexicons for non-work related activities, allowed us to straightforwardly assign a MET value using the Compendium. Although the Compendium includes a list of occupations with specific MET values, the list is not comprehensive and is not compatible with most international standard occupational classification systems such as the International Standard Classification of Occupations (ISCO) [24] and Standard Occupational Classification (SOC) [25]. Therefore, it is difficult to link the occupational information included in household and individual questionnaires in time-use surveys (collected alongside the diary data) with the occupations listed in the Compendium, as these are usually coded using ISCO or SOC. A number of studies report different approaches to measuring OA [10, 26, 27], but very few published studies link MET values to detailed occupational classification systems. Below, we describe the two published studies linking MET values to SOC or similar classifications using time-use data.

In the first study, Tudor-Locke and colleagues [11, 28] used the occupational category variables present in the time-use survey questionnaire data for each working respondent, and assigned MET values using the Tecumseh Occupational Physical Activity Questionnaire (TOPAQ) classification system. This system incorporates body position (sit, stand, walk, heavy effort) and activity intensity (light, moderate and vigorous) when assigning MET values to specific occupations. Following this approach, they attached MET values to the 22 major occupational groups present in the data, as well as to the 509 detailed occupations within the 2002 Census Occupational Classification System [11, 28]. In the second study, Spinney, Millward and Scott attached MET values to the most general level of the Canadian SOC and the Standard Industry Codes (SIC) in a study assessing population-level changes (1992, 1998 and 2005) in the proportion of ‘active living’ Canadian adults. First, they classified all relevant occupational codes from the Compendium into general occupation sectors present in their data. Second, they assigned the median MET value of these occupational Compendium codes to the broad occupational categories in their own data [22].

The Compendium was a useful resource when planning how to assign METs to occupations, particularly when considering jobs involving different levels of PAEE across the day (e.g. manual labour). However, we could not find any published replicable method linking METs to widely used standard occupational classification systems. Furthermore, some occupations listed in the Compendium (e.g. labourers using heavy tools, code 11790) were assigned very high MET values, which does not take into account periods of job-specific tasks with lower PAEE. Whilst actively using heavy machinery may involve vigorous activity at 8 METs, twirling the ‘stop’ and ‘go’ sign, or conferring at the roadworks site would not. Sedentary occupations (e.g. clerks, bus drivers) are less problematic, as the METs would likely only range from 1.3 to 1.5.

One of the difficulties with the METs calculations reported by Tudor-Locke [11, 28] and Spinney and their colleagues [22] is that the procedures they followed were rather vague and therefore difficult to replicate. The degree of detail and variation in the MET values assigned to the general occupation codes in Tudor-Locke’s and Spinney’s calculations is rather small. Given that we cannot identify different job-specific tasks during paid work from the diary data, we are convinced that it is prudent to use the most detailed occupational information available when assigning MET values to specific occupations. Broad categorisations comprising several specific occupations are likely to result in under- or overestimations of OA.

### An alternative approach to applying MET values to occupational codes

In the process of developing a procedure for assigning MET values to standard occupational classification systems, we considered it important to be as explicit and transparent as possible to allow other researchers to replicate, adapt and adjust our calculations. To achieve our goal, we documented the full procedure, which we now outline.

#### Applying METs to standard occupational classification systems

The 2008 International Standard Classification of Occupations (ISCO-08) manual [24] provided us with a complete overview of all occupations, together with a list-wise detailed description of all job-specific tasks attached to those occupations. Therefore, the ISCO-08 occupational classification system – given that it can be straightforwardly linked to the occupational codes in the associated individual and household questionnaires in time-use surveys – served as a logical starting point for the first stage of our coding procedure. The ISCO-08 is designed in such a way that occupations worldwide can be assigned to one of the ‘Unit Groups’. This classification structure is hierarchical and clusters occupations with a high degree of similarity in terms of skill levels and specialisations: ‘Unit Groups’ are grouped into ‘Minor Groups’, which are clustered into ‘Sub-Major groups’, which are grouped into ‘Major Groups’. This results in a four-digit code, with each of the four numbers referring to this hierarchical structure [24]. Table 1 illustrates this structure for university and higher education teachers (ISCO-08 code 2310) [24].

**Table 1:** Structure of ISCO-08 Occupational Codes, example for code 2310 ‘University and higher education teachers’

Hierarchical structure	Coding and ‘label’
Major Group	2 ‘Professionals’
Sub-Major group	23 ‘Teaching professionals’
Minor Group	231 ‘University and Higher Education Teachers’
Unit Group	2310 ‘University and Higher Education Teachers’

After examining the ISCO-08 manual [24], we realised that we needed to include as much detail as possible in our procedure, given the variety of job-specific tasks of occupations belonging to broader occupational categories. The occupations listed in the Compendium are not divided into specific tasks, and recognising that certain types of occupations involve tasks ranging from light to vigorous PA, we considered it important to code with the highest-possible levels of consistency and reliability.

We first generated a detailed spreadsheet of all occupations and their job-specific tasks to the four-digit-level based on the ISCO-08 manual. We achieved this by copying the bulleted list of all job-specific tasks associated with each of the 436 different occupations or ‘Unit Groups’ in this document [24].

Next, we attached a MET value to each of the job-specific tasks for every occupation or ‘Unit Group’. We used many of the ‘Volunteer Activity’, ‘Home Activity’, and ‘Occupation’ activity categories from the Compendium in the coding process (e.g. Compendium codes 21,015 ‘standing, light work (filing, talking, assembling)’ 2.3 METs; 5052 ‘cooking or food preparation, walking’ 2.5 METs and; 11,580 ‘sitting tasks, light effort (e.g., office work, chemistry lab work, computer work, light assembly repair, watch repair, reading, desk work)’ 1.5 METs). However, we soon discovered that there were many similar job-specific tasks – in terms of content and PAEE – across occupations that were listed in the Compendium (e.g. the job-specific tasks ‘designing and modifying curricula and preparing courses of study in accordance with requirements’ of the university and higher education teachers’ (ISCO-08 code 2310) and ‘planning and organizing individual and group activities designed to facilitate the development of children’s motor, cooperative and social skills, confidence and understanding’ of early childhood educators (ISCO-08 code 2342)).

At this stage, we began to develop a list of tasks (hereafter ‘task abbreviations’) comparable across a number of occupations (e.g. both job-specific tasks of the teaching professionals from the previous example were abbreviated as ‘designing curriculum/lessons/activities’ and assigned a MET value of 1.3 using the Compendium code 9060 ‘sitting, studying, general, including reading and/or writing, light effort’). Constructing the task abbreviation list was an iterative process, as some of the tasks became confusing when applied to different types of occupations (e.g. the task ‘informing, promoting and interviewing customers, suppliers, or employees’ that was used in occupations including social work and counselling professionals (ISCO-08 code 2635), or insurance representatives (ISCO-08 code 3321) and was assigned a MET value of 1.5 using the Compendium code 9055 ‘sitting, talking in person, on the phone, computer, or text messaging, light effort’). As we progressed through the various occupational categories, we adjusted the task abbreviations accordingly.

Parallel to this process, we established a procedure for allocating METs to occupations or activities not listed in the Compendium. Many of the tasks involved the basic positions of sitting, standing, walking, lifting and climbing – with talking adding about 0.2 METs (e.g. Compendium code 9055 ‘sitting, talking in person, on the phone, computer, or text messaging, light effort’ 1.5 METs and 9060 ‘sitting, studying, general, including reading and/or writing, light effort’ 1.3 METs). We used these ‘task component calculations’ to help us calculate appropriate MET values for the ‘unlisted’ job-specific tasks.

#### Assigning MET values to ISCO-08 occupation codes.

We used either or both the task ‘abbreviations’ and ‘component calculations’ to attach a MET value to each job-specific task of every occupation listed in the ISCO-08 manual. We argue that even if the MET assignments are not completely accurate, we can account for every step in the process, which enables other researchers to adjust or adapt the process we report. As our final measure for this stage of the project, we calculated a MET value for each of the 436 different occupations or ‘Unit Groups’ in ISCO-08 by averaging the job-specific MET values belonging to each occupation. This calculation procedure is illustrated in Tables 2 and 3. Given the present state of knowledge about OA we believe that our procedure can identify areas for further in-depth OA research, and can be used as an add-on to research into PAEE and OA using time-use and other survey data. Using the ISCO (2008) to the UK SOC (2010) crosswalk [25], our calculations can also be attached to the UK and other international SOC codes (e.g. US, Canada and Australia).

**Table 2:** Coding example for university and higher education teachers (ISCO-08 code 2310)

Job-specific tasks from the ISCO-8 job classification document	Task abbreviations	Compendium code and label	MET value
(a) designing and modifying curricula and preparing courses of study in accordance with requirements	Designing curriculum, lessons and activities	9060 ‘sitting, studying, general, including reading and/or writing, light effort’	1.3
(b) preparing and delivering lectures and conducting tutorials, seminars and laboratory experiments	Delivering lectures	11,791 ‘walking on job, less than 2.0 mph, very slow speed, in office or lab area’	2
(c) stimulating discussion and independent thought among students	Stimulating discussion	11,791 ‘walking on job, less than 2.0 mph, very slow speed, in office or lab area’	2
(d) supervising, where appropriate, experimental and practical work undertaken by students	Supervising of students, staff and colleagues	11,791 ‘walking on job, less than 2.0 mph, very slow speed, in office or lab area’	2
(e) administering, evaluating and marking examination papers and tests	Marking papers and tests	9040 ‘sitting, writing, desk work, typing’	1.3
(f) directing research of post-graduate students or other members of department	Directing and participating in research	11,585 ‘sitting meetings, light effort, general, and/or with talking involved’	1.5
(g) researching into and developing concepts, theories and operational methods for application in industrial and other fields	Desk based research	9040 ‘sitting, writing, desk work, typing’	1.3
(h) preparing scholarly books, papers or articles	Preparing papers	9040 ‘sitting, writing, desk work, typing’	1.3
(i) participating in departmental and faculty meetings and in conferences and seminars	Meetings	11,585 ‘sitting meetings, light effort, general, and/or with talking involved’	1.5
		MET value for ISCO code 2310	1.58
		Standard deviation	0.327

**Table 3:** Coding example for early childhood educators (ISCO-08 code 2342)

Job-specific tasks from the ISCO-8 job classification document	Task abbreviations	Compendium code and label	MET value
(a) planning and organizing individual and group activities designed to facilitate the development of children’s motor, cooperative and social skills, confidence and understanding	Designing curriculum, lessons and activities	9060 ‘sitting, studying, general, including reading and/or writing, light effort’	1.3
(b) promoting language development through storytelling, role play, songs, rhymes and informal conversations and discussions	Teaching kindergarten (3 – 6 years)	21,017 ‘standing, child care, only active periods’	3
(c) leading children in activities that provide opportunities for creative expression through the media of art, dramatic play, music and physical fitness	Teaching kindergarten (3 – 6 years)	21,017 ‘standing, child care, only active periods’	3
(d) observing children in order to evaluate progress and to detect signs of developmental, emotional or health-related problems	Observing and evaluating students	11,791 ‘walking on job, less than 2.0 mph, very slow speed, in office or lab area’	2
(e) observing and assessing nutritional health, welfare and safety needs of students and identifying factors which may impede students’ progress	Observing and evaluating students	11,791 ‘walking on job, less than 2.0 mph, very slow speed, in office or lab area’	2
(f) supervising children’s activities to ensure safety and resolve conflicts	Supervising of students, staff and colleagues	11,791 ‘walking on job, less than 2.0 mph, very slow speed, in office or lab area’	2
(g) guiding and assisting children in the development of proper eating, dressing and toilet habits	Teaching kindergarten (3 – 6 years)	21,017 ‘standing, child care, only active periods’	3
(h) discussing progress or problems of children with parents and other staff members and identifying appropriate actions and referrals to other services	Discussion student reports	11,585 ‘sitting meetings, light effort, general, and/or with talking involved (e.g., eating at a business meeting)’	1.5
(i) establishing and maintaining collaborative relationships with other service providers working with young children	Networking	11,791 ‘walking on job, less than 2.0 mph, very slow speed, in office or lab area’	2
		MET value for ISCO code 2310	2.2
		Standard deviation	0.65

#### Statistical analysis

In addition to calculating a mean MET value per occupation in ISCO-08, we calculated the associated standard errors of the job-specific MET values per occupation. Finally, we averaged the mean MET values and standard deviations of all occupations belonging to the Major Groups in the ISCO-08 manual. This last aggregated measure on the level of the Major Groups serves as a measure of dispersion which we use in the next section to test our coding procedure. Tables 2 and 3 illustrate these calculations.

## Results

### Testing the procedure to identify areas for refinement

Our approach can best be explained by comparing the assignment of MET values to underlying job-specific tasks for two occupations within the same ISCO-08 Sub-Major group of ‘teaching professionals’. Tables 2 and 3 illustrate the coding procedure for university and higher education teachers (ISCO-08 code 2310) and early childhood educators (ISCO-08 code 2342). Whilst the job specific tasks are quite similar, the working conditions are different (e.g. student age, proportion of face-to-face teaching hours to teaching preparation, subjects taught) which is likely to affect the expected PAEE and subsequent assignment of MET values. Given that the job-specific task of the average early childhood educator includes more tasks that involve walking on the job than university and higher education teachers, their average MET value is higher than the latter.

**Fig. 2:**
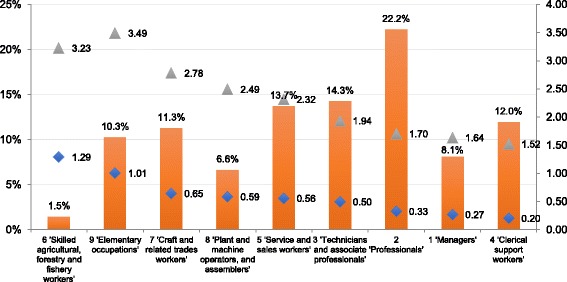
Mean standard deviations and average MET values for all ISCO level 1 occupational categories. *Square orange* Percentages workers Belgian population (LFS13). *Diamond Blue* Mean standard deviations. *Gray Triangle* Mean METs

Having established that occupations involving job-specific tasks primarily within the ‘sedentary’ and lower end of ‘light’ PAEE ranges (i.e. 1.3–1.6 METs) are unlikely to be subject to serious over- or under-estimation, we turned our attention to less straightforward occupations. It is clear that occupations involving job-specific tasks with wider ranges of PAEE are more difficult to classify in terms of their METs expenditure (e.g. unskilled labourers). So, the next stage in our process was to identify those occupations at greatest risk of over or underestimation when applying our procedure Fig. 2.

This figure clearly indicates that ‘clerical support workers’, ‘managers’, and ‘professionals’ work in occupations with fairly low mean MET values and low mean standard deviations, which indicates that there is little PAEE variation in the underlying job-specific tasks. For ‘technicians and associate professionals’, ‘service and sales workers’ and ‘plant and machine operators and assemblers’ we see somewhat higher mean standard deviations and mean MET values. It is within the groups of ‘craft and related trades workers’, ‘elementary occupations’ and ‘skilled agricultural, forestry and fishery workers’ that we see the highest mean MET values and mean standard deviations, occupations that involve job-specific tasks ranging from vigorous to light – an explanation for the wider dispersion that needs to be investigated more thoroughly.

Based on a closer inspection of the mean METs and the dispersion measure, we recommend prioritising the ‘elementary occupations’ and ‘craft and related trades workers’ for further in-depth research into OA. In 2013, 21.6% of the Belgian working population were employed in occupations within these two Major Groups. These occupations are at greatest risk of over or underestimation when applying our procedure because of their high mean METs and dispersion measure.

In order to stimulate debate and identify areas for further research, we attached all our calculations in a spreadsheet [see Additional file 1].

## Discussion

Given that time-use data and the associated individual and household questionnaires provide rich contextual information, they are well suited for analysing PA using large representative samples in a way that has not been carried out in self-report or device-based PA studies. Time-use respondents record their daily activities in a paper or online diary in 10-min intervals across 24-h (1440 min). The continuous recording of activities reduces problems of social desirability because diarists cannot prolong certain socially-valued activities (e.g. exercising) without reducing another (e.g. watching television), as all activities must sum to 24 h. Furthermore, time-use surveys are generally multi-purpose (i.e. the data are used by researchers from a number of disciplines for a range of purposes), so there is no explicit focus, other than the accurate recording of people’s daily activities. Generally, people do not naturally aggregate the time they spend in different activities, so continuous recording reduces errors of recall and over- and underestimation of time allocated to different activities [14].

Although most time-use studies provide detailed data on unpaid work (e.g. child and elder care, housework, meal preparation), leisure, travel and sleep, respondents only record their total working hours and any coffee or lunch breaks during the working day. In order to make time-use surveys suitable for PA research, and lacking specific data on job-specific tasks during work episodes, we developed a procedure for assigning MET values to paid work durations recorded in the diaries. We used the detailed occupational data included in the associated individual and household questionnaires to calculate OA. Although several researchers have attempted to quantify OA [10, 26], we found only two studies that used detailed international occupational classification systems (e.g. ISCO or SOC) to calculate OA [11, 22, 28].

Because the reported procedures were difficult to replicate, we developed and documented an alternative and more transparent procedure that other researchers could replicate. We also wanted to present our work as a stimulus for further research into OA. We used the ISCO-08 manual as a starting point because it includes all possible occupations (or occupational groups) in developed countries and delivers a detailed overview – in the format of a bullet-point list – of all job-specific tasks associated with these occupations [24]. This list, combined with the Compendium, formed the basis for our assignment of MET values to all occupational codes in the ISCO-08 manual. We acknowledge that occupations – and their underlying job-specific tasks – are changing as many jobs become more automated and computer-assisted. The procedure we recommend is able to accommodate these changes, as MET values for all occupations in the ISCO-08 manual [24] are calculated using the basic elements of sitting, standing, walking, or lifting in the underlying job-specific tasks. If some underlying job-specific tasks change – and assuming that increasing levels of automation are likely to result in reduced levels of PA or even increased SB – our procedure can accommodate these changes.

We acknowledge that our calculations – simply averaging all MET values assigned to the job-specific tasks for a given occupation – are well-documented but nevertheless ‘educated guesses’, and may not reflect actual OA expenditures. In our procedure, we assigned the same weight to each job-specific task, and so could not take into account the relative proportion of time individuals spent in various job-specific tasks. Whilst we are confident that our OA calculations for light or sedentary occupations are reliable and valid, we acknowledge that occupations involving moderate to vigorous physical activity (MVPA) and those occupations with wide variations in METs expenditures of their underlying job-specific tasks require more careful analysis.

## Conclusions

Whilst the life domain-based approach frequently used in PA research is informative, time-use data allow researchers to examine respondents’ daily activities within the 24-h context, including co-presence, location or mode of transport, and secondary/simultaneous activities. By using whole-day data the associations between life domains can be better understood (e.g. the relationship between OA and LTPA), and the proportion of PAEE within and between domains estimated. We invite other researchers to discuss and investigate – in terms of the dispersion of the underlying job-specific MET values for specific occupations – more complex occupations in order to produce improved and objectively-measured OA estimates that can be linked to the occupational classification system in existing databases. This could be achieved by carrying out systematic observations of specific jobs, undertaking documentary analysis (e.g. examining work schedules or specific job descriptions), studying industrial regulations (e.g. the maximum hours allowed to work in certain tasks) or by conducting fieldwork for specific occupations (e.g. observing small samples of workers). An example of such an approach is the CAPTURE-24 study, where respondents wore an accelerometer and a wearable camera for one day while they recorded their time-use in a self-report time-use diary [29]. This combination of time-use data and images, together with objectively-measured PA, yields in-depth contextual data about the job-specific tasks and the proportion of time workers spend in these tasks throughout a working day.

We acknowledge that precise estimates of OA are not possible until we have objective data on the proportion of time people spend in various job-specific tasks, taking regional differences into account. Inspired by and building on the work of Ainsworth, Tudor-Locke, and Spinney and their colleagues [11, 15, 16, 22, 23, 28], we view our calculations as a methodological and pragmatic contribution to future investigation into the objective measurement of OA using standard occupational classification systems.

## Additional file

Additional file 1: This file contains – in its separate tabs – the different steps of the calculation procedure we outlined in paragraph *‘4.1 An alternative approach to applying MET values to occupational codes’*. The *first tab ‘ISCO - METs calculation’* provides a list of all 436 four-digit ISCO-08 codes and their underlying job-specific tasks. We assigned MET values to these underlying job-specific tasks using our ‘task abbreviations/task component calculations’ list, which we present in the *second tab ‘Job-specific tasks’*. The METs assigned to this list were based on the most recent version of the Ainsworth compendium [23], which we outlined in the *third tab ‘Compendium - Ainsworth (2011)’*. The *fourth and final tab ‘ISCO - METs table’* presents an overview of all four-digit ISCO-08 codes and their assigned MET values. (XLSX 522 kb)

## Abbreviations

ATUS: American Time-Use Survey
GPAQ: Global physical activity questionnaire
GPS: Global positioning systems
HETUS: Harmonized European Time-Use Survey
IPAQ: International physical activity questionnaire
ISCO-08: International Standard Classification of Occupations
LFS: Labour Force Survey
LTPA: Leisure time physical activity
METs: Metabolic Equivalent of Task
MTUS: Multinational Time-Use Study
MVPA: Moderate to vigorous physical activity
OA: Occupational activity
PA: Physical activity
PAEE: Physical activity energy expenditure
SB: Sedentary behaviour
SIC: Standard Industry Codes
SOC: Standard Occupational Classification
TOPAQ: Tecumseh Occupational Physical Activity Questionnaire
